# Maternal Synchronization of Gestational Length and Lung Maturation

**DOI:** 10.1371/journal.pone.0026682

**Published:** 2011-11-09

**Authors:** Valérie Besnard, Susan E. Wert, Machiko Ikegami, Yan Xu, Caleb Heffner, Stephen A. Murray, Leah Rae Donahue, Jeffrey A. Whitsett

**Affiliations:** 1 The Perinatal Institute and Section of Neonatology, Perinatal and Pulmonary Biology, Cincinnati Children's Hospital Medical Center, the Department of Pediatrics and The University of Cincinnati College of Medicine, Cincinnati, Ohio, United States of America; 2 The Jackson Laboratory, Bar Harbor, Maine, United States of America; University of Tübingen, Germany

## Abstract

Among all mammals, fetal growth and organ maturation must be precisely synchronized with gestational length to optimize survival at birth. Lack of pulmonary maturation is the major cause of infant mortality in preterm birth. Whether fetal or maternal genotypes influence the close relationship between the length of gestation and lung function at birth is unknown. Structural and biochemical indicators of pulmonary maturity were measured in two mouse strains whose gestational length differed by one day. Shorter gestation in C57BL/6J mice was associated with advanced morphological and biochemical pulmonary development and better perinatal survival when compared to A/J pups born prematurely. After ovarian transplantation, A/J pups were born early in C57BL/6J dams and survived after birth, consistent with maternal control gestational length. Expression of genes critical for perinatal lung function was assessed in A/J pups born after ovarian transfer. A subset of mRNAs important for perinatal respiratory adaptation was selectively induced in the A/J pups born after ovarian transfer. mRNAs precociously induced after ovarian transfer indicated an important role for the transcription factors C/EBPα and CREB in maternally induced lung maturation. We conclude that fetal lung maturation is determined by both fetal and maternal genotypes. Ovarian transfer experiments demonstrated that maternal genotype determines the timing of birth and can influence fetal lung growth and maturation to ensure perinatal survival.

## Introduction

Premature birth is associated with impaired maturation and function of various organs, limiting perinatal adaptation. Pulmonary immaturity and associated deficiency of pulmonary surfactant are the major factors affecting survival of preterm infants worldwide [1]. Preterm birth rates and associated pulmonary morbidities have increased progressively during recent decades in North America [2], [3]. While obstetrical therapies used to prevent preterm deliveries have been unsuccessful, therapies designed to delay delivery, providing time for maternal treatment with glucocorticoids to enhance fetal lung maturation, have been useful in the prevention of respiratory distress syndrome (RDS) in preterm infants, supporting the importance of lung maturation in the transition to air breathing at birth [4].

RDS in preterm infants is caused by the lack of pulmonary surfactant, which is required to reduce surface tension at the air-liquid interface in the alveolus after birth. In preterm infants, lack of maturation of lung structure also contributes to respiratory dysfunction. Differentiation of respiratory epithelial cells lining the peripheral lung increases in later stages of lung morphogenesis. Lung maturation is associated with 1) expansion of peripheral lung saccules and the formation of septae to produce alveoli, 2) production and secretion of pulmonary surfactant required to reduce surface tension at the air-liquid interface after birth, 3) clearance of lung fluids after birth, and 4) thinning of the alveolar walls and the expansion of the pulmonary vascular bed to facilitate gas exchange across the alveolar capillary barrier [5]. Lung morphogenesis and maturation are regulated by signaling and transcriptional networks that influence gene expression, cell proliferation, and differentiation [6–8 for review]. Mechanisms by which fetal organ maturation and gestational length are integrated to ensure neonatal survival remain poorly understood. We asked whether the length of gestation was synchronized with lung maturation and whether lung maturity was determined by maternal or fetal genotype.

## Results

### Gestational length, body weight and lung weight differ in C57BL/6J and A/J mice

**Figure 1:**
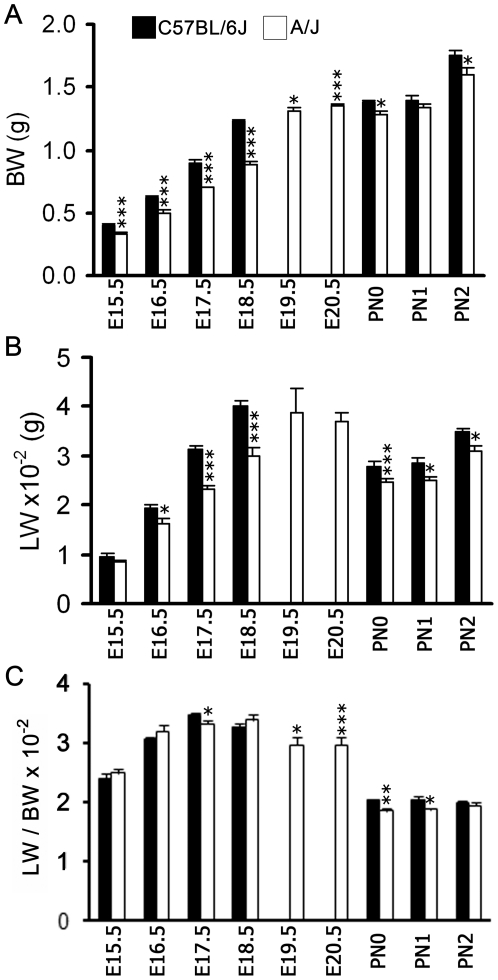
Advanced lung and somatic fetal growth in C57BL/6J (parturition = E19.5) compared to A/J (parturition = 20.5) fetuses. Body weight (A), lung weight (B), and lung-to-body weight ratios (C) of C57BL/6J and A/J mice were measured from fetuses harvested at E15.5, E16.5, E17.5, E18.5, E19.5 (A/J), and E20.5 (A/J), and pups at PN0, PN1 and PN2. Prior to birth, data obtained at E19.5 and E20.5 in the A/J strain were compared to E18.5 data in the C57BL/6J strain. Values are means ± SEM of 12 animals per strain; *p< 0.05, **p< 0.01, ***p< 0.001 for A/J vs. C57BL/6J.

Relationships between gestational length and fetal lung maturation were assessed in C57BL/6J and A/J mice. While C57BL/6J dams gave birth on embryonic (E) day 19.5±0.9 hr, A/J dams delivered pups on E20.5±1 hr as recently reported [9]. Body weight (BW) and lung wet weight (LW) were higher in C57BL/6J than in A/J fetuses at identical gestational times (Figure 1A, 1B). During additional gestational days in the A/J strain (E19.5 and E20.5 just prior to birth), BW and LW in the A/J fetuses were similar to C57BL/6J fetuses at E18.5. The lung-to-body weight (LW/BW) ratio was reduced modestly in both strains from either E18.5 (C57BL/6J) or E19.5 (A/J) to PN0, consistent with loss of fetal lung fluid after birth (Figure 1C). Growth velocity was lower in A/J mice (slope = 0.1812±0.01729) compared to C57BL/6J mice (slope = 0.2152±0.006298) (p<0.0001) in late gestation. Thus, between E15.5 and E18.5, both body and lung weight increased earlier in the C57BL/6J mice. By the time of birth (PN0), lung weight was similar in both strains (Figure 1A).

### Structural maturation of the lung occurs earlier in C57BL/6J mice

**Figure 2:**
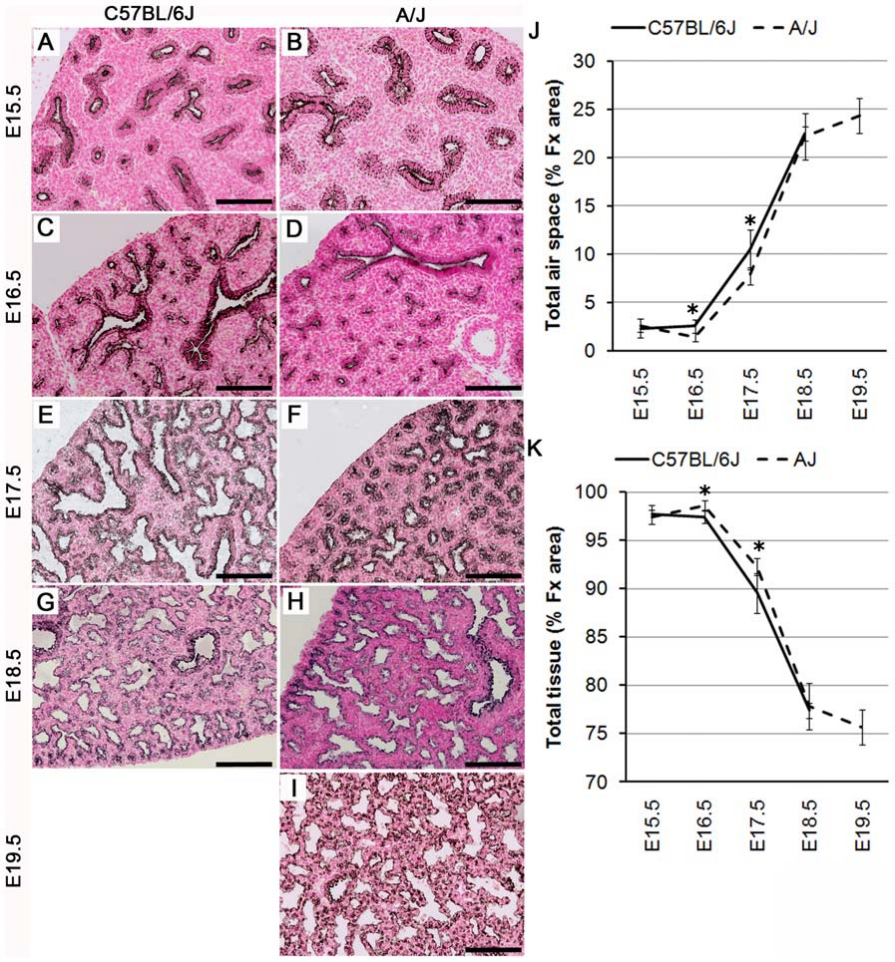
Advanced lung sacculation in C57BL/6J fetal mice. Pancytokeratin was used to identify respiratory epithelia in C57BL/6J (A, C, E, G) and A/J (B, D, F, H, I) lungs from fetuses harvested at E15.5 (A, B), E16.5 (C, D), E17.5 (E, F), E18.5 (G, H), E19.5 (I) (A/J only). At E16.5 and E17.5, lung maturation was more advanced in C57BL/6J fetuses compared to A/J at identical gestational days. Note the thinner walls and dilatation of peripheral saccules in C57BL/6J compared to A/J fetuses. Scale bar is 100 µm. Changes in fractional areas (% Fx area) of total airspace (J) and tissue compartment (K) were determined in C57BL/6J and A/J embryonic lungs harvested at E15.5, E16.5, E17.5, E18.5, and E19.5 (A/J only). Values are means ± SEM of 4 animals per strain; *p< 0.05 for A/J vs. C57BL/6J.

During the perinatal period, the lung undergoes dramatic structural changes required for respiration at birth. Peripheral lung saccules dilate, the mesenchyme thins, pulmonary vascularity increases, and the epithelium differentiates to produce cuboidal alveolar type II and squamous alveolar type I cells. Dilation of the peripheral lung parenchyma at E16.5 and E17.5 was delayed in A/J compared to C57BL/6J mice (Figure 2A–H). During additional days of gestation, sacculation of A/J mice matured to that of the C57BL/6J mice (Figure 2I). Morphometric analysis demonstrated that the relative proportion, or fractional area (% Fx area), of air space at E16.5 and E17.5 in the lungs of the A/J mice was significantly less than in the C57BL/6J mice (Figure 2J–K). The luminal area of the peripheral saccules increased earlier in C57BL/6J fetuses compared to that in the A/J fetuses, consistent with earlier onset of this aspect of lung maturation in the C57BL/6J strain. At E18.5, the morphological maturity of lungs from A/J and C57BL/6J fetuses was similar (Figure 2G–H).

### Strain differences in surfactant production in C57BL/6J and A/J mice

**Figure 3:**
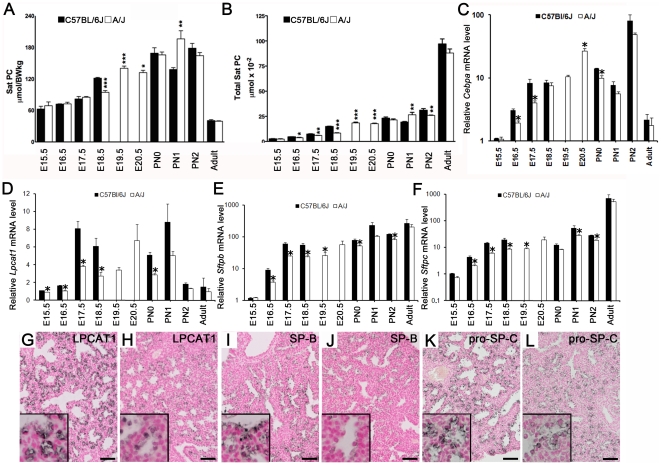
Earlier induction of SatPC, surfactant associated mRNAs and protein in lungs of C57BL/6J fetal mice. SatPC was measured in lungs from C57BL/6J and A/J fetuses harvested at E15.5, E16.5, E17.5, E18.5, E19.5 and E20.5 (A/J only), and pups at PN0, PN1 and PN2 (A–B). Lung SatPC content was significantly increased in C57BL/6J compared to A/J mice at identical gestational days. At E19.5 and E20.5, lung SatPC pool sizes in A/J fetuses were similar to those on E18.5 in the C57BL/6J strain. Results are expressed as the means ± SEM of 12 animals per strain; *p< 0.05, **p<0.01, ***p<0.01 for A/J vs. C57BL/6J. mRNAs for *Cebpa* (C), *Lpcat1* (D), *Sftpb* (E), and *Sftpc* (F) were assessed by quantitative RT-PCR in lungs of C57BL/6J and A/J mice and normalized to 18S rRNA. Values are means ± SEM; *p< 0.05 for A/J vs. C57BL/6J. Lung sections for C57BL/6J (G, I, K) and A/J (H, J, L) obtained at E17.5 were stained for LPCAT-1 (G, H), mature SP-B (I, J) and pro-SP-C (K, L). Staining for LPCAT-1, SP-B and pro-SP-C was increased in C57BL/6J compared to A/J fetuses. Prior to birth, data obtained at E19.5 and E20.5 in the A/J strain were compared to E18.5 data in the C57BL/6J strain. Photomicrographs are representative of 4 individual fetal mice. Scale bar is 50 µm; inset magnification is increased by 3.2.

Surfactant reduces surface tension at the air-liquid interface and maintains lung volumes required for lung function after birth. Surfactant function in the lung depends on synthesis of both saturated phosphatidylcholine (SatPC) and surfactant associated proteins. Fetal lung SatPC, a critical component of surfactant, increased with advancing gestation in both strains of mice, increasing earlier in C57BL/6J fetuses (Figure 3A–B). Likewise, mRNAs encoding proteins critical for postnatal lung function and surfactant homeostasis, including *Cebpa*, *Lpcat1*, *Sftpb*, and *Sftpc* increased in both strains from E15.5 to E18.5 (Figure 3C–F). In relation to gestational length, *Cebpa, Lpcat1*, *Sftpb,* and *Sftpc* mRNAs increased earlier in C57BL/6J than in A/J mice (Figure 3C–F). Similarly, immunohistochemical staining for LPCAT1, SP-B, and pro-SP-C was detected earlier in lung sections from the C57BL/6J mice (Figure 3G–L). The increase in expression of SP-B and LPCAT1 with advancing gestation and their requirement for surfactant function at birth [10], [11], are consistent with earlier lung maturation in the C57BL/6J strain. The prenatal increase in mRNA expression, as well as changes in the tissue distribution of C/EBPα, a transcription factor required for normal surfactant protein and lipid production and for lung function in late gestation [12], [13], also occurred earlier in C57BL/6J mice (Figure 3C, Figure S1).

### Expression of lung mRNAs associated with lung maturation and transition to air breathing

**Figure 4:**
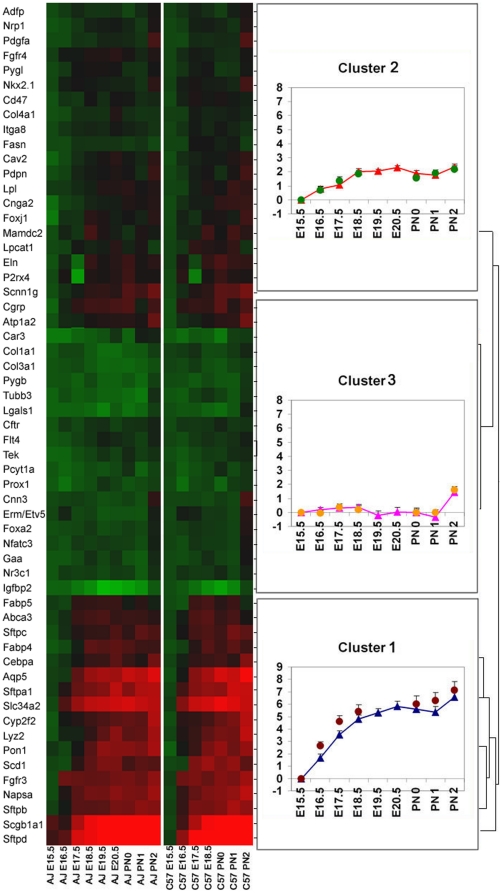
Strain and length of gestation influence gene expression in the developing lung. mRNA levels encoding 53 proteins associated with lung maturation were measured by qRTPCR. mRNAs were clustered hierarchically into three distinct groups. The heat map (left panel) was generated using log2-transformed data and complete linkage to calculate distance between clusters. The mRNA profile charts (right panels) of three clusters of genes were generated based on normalized mean ± SEM within each gene cluster. Triangles and circles represent the normalized group mean expression value of A/J and C57BL/6J mice, respectively, at each gestational time point. The X-axis represents gestational time points. The Y-axis represents relative expression normalized to the E15.5 value of each respective strain.

Developmental changes in lung mRNAs were compared in C57BL/6J and A/J mice, assessing the levels of 53 mRNAs previously associated with lung function and structure at each gestational time point and after birth. Hierarchical clustering of the time-dependent changes in mRNAs identified distinct subgroups of genes that were coordinately expressed across developmental stages (Figure 4). The mRNAs were grouped into three distinct clusters. mRNAs in “Cluster 1,” including those encoding surfactant associated proteins *Sftpa1, Sftpb, Sftpc, Sftpd, Abca3,* and *Slc34a2*, increased markedly with advancing gestation in both strains, increasing earlier in C57BL/6J fetuses. In contrast, expression of those mRNAs in “Cluster 2,” including *Nkx2.1, Pdgfa,* and *Lpcat1*, increased moderately across gestational ages in both strains. mRNAs in “Cluster 3,” including *Foxa2, Nfatc3, Nr3c1*, and *Etv5*, transcription factors known to be important transcriptional regulators of lung maturation, did not change substantially with advancing gestation, supporting the concept that their activity is not mediated primarily by the level of their transcripts but perhaps by post-transcriptional mechanisms. Consistent with these findings, immunohistochemical staining of FOXA2 was similar in respiratory epithelial cells in both strains (Figure S2). Bronchi and bronchioles of murine lungs are lined by pseudostratified or simple columnar epithelium composed of basal, ciliated, Clara, and goblet cells that together mediate barrier function, mucociliary clearance, and innate host defense, vital for pulmonary homeostasis. Maturational markers of conducting airway epithelial cell differentiation, Clara cell secretory protein (CCSP or SCGB1A1) and acetylated α-tubulin, increased earlier in C57BL/6J fetuses (Figures S3 and S4). Taken together, these findings indicate that the shorter gestational length in C57BL/6J mice was associated with earlier maturation of the respiratory epithelium lining both proximal and peripheral airways. Using multivariate correlation analysis of mRNA expression with body weight, lung weight, SatPC and morphometric measurements, we identified a subset of mRNAs, including *Sftpa, Sftpb, Sftpc, Sftpd, Slc34a2, Scgb1a1*, *Cebpa* and *Aqp5*, that correlated well with SatPC, body weight, lung weight, and the fractional area of airspace (Figure S5). Likewise, genes associated with lipid homeostasis, including *Scd1, Abca3, Fabp5*, and *Lpcat1*, were correlated with both lung weight and fractional area of airspace (Figure S5); while another distinct subset of mRNAs, including *Tubb3*, *Pygb*, and *Igfbp2*, were correlated with fractional area of the tissue compartment (Figure S5). Thus, expression of a subset of mRNAs enriched in proteins involved in surfactant homeostasis was highly correlated with increasing SatPC, body weight, lung weight, and structural maturation of the lung as gestation proceeded.

### Maternal control of gestational length

To determine whether gestational length depends on the genetic background of the mother or the fetus, we performed ovarian transfer experiments. C57BL/6J SCID mice received A/J ovaries and were then fertilized by A/J males. In spite of bearing A/J fetuses, C57BL/6J SCID mice gave birth at E19.5, as expected for the C57BL/6J strain and in contrast to the typical gestational length of 20.5 days for the A/J strain [9]. Likewise, when C57BL/6J ovaries were transplanted into C57BL/6J SCID mice, gestational length was 19.5 days, as expected from the maternal strain [9]. Thus, the maternal genetic background, not that of the fetus, controlled the timing of normal parturition in these two strains of mice. C57BL/6J SCID mice receiving either C57BL/6J SCID ovaries (B6-OT) or A/J ovaries (AJ-OT) produced litters of similar size (5.2±0.8 B6-OT pups/litter, n = 5 litters vs. 4.8±1.1 AJ-OT pups/litter, n = 5 litters, p = 0.67), all of which survived after birth. AJ-OT mice had similar BW and significantly higher LW and LW/BW ratios compared to B6-OT, indicating that the C57BL/6J maternal environment had a positive effect on lung growth of the A/J pups (Figure S6A-C). SatPC levels were similar in B6-OT, AJ-OT, and A/J lungs at E17.5 and PN1 (Figure S6D-E). Reciprocal ovarian transfer experiments were not performed due to the lack of A/J SCID mice or similarly immuno-compromised strains needed for the experiment.

Both perinatal growth and lung maturation were sufficient for survival of AJ-OT mice when born one day earlier to C57BL/6J SCID dams (parturition 19.5 days). When A/J fetuses from A/J dams (parturition 20.5 days) were delivered two days prematurely at E18.5 by hysterectomy, all A/J pups (n = 27) failed to expand their lungs, became cyanotic and died of respiratory failure soon after birth. However, A/J fetuses delivered one day prematurely at E19.5 from A/J dams maintained oxygenation and survived (survival rate = 87.4%±4.6, n = 26), indicating that only one additional gestational day was required for functional maturation of the lungs of A/J fetuses. Likewise, when C57BL/6J mice were born one day prematurely at E18.5 from C57BL/6J dams, most of the pups initiated respiration, became oxygenated and survived (survival rate = 82.5%±6.8, n = 37). Taken together, these data support the concept that maternal-fetal interactions, determined by the maternal genome, influence the length of the normal gestational period and that the maternal environment influences lung maturation in these mouse strains.

### Distinct aspects of fetal lung maturation are controlled by maternal and fetal genotypes

**Figure 5:**
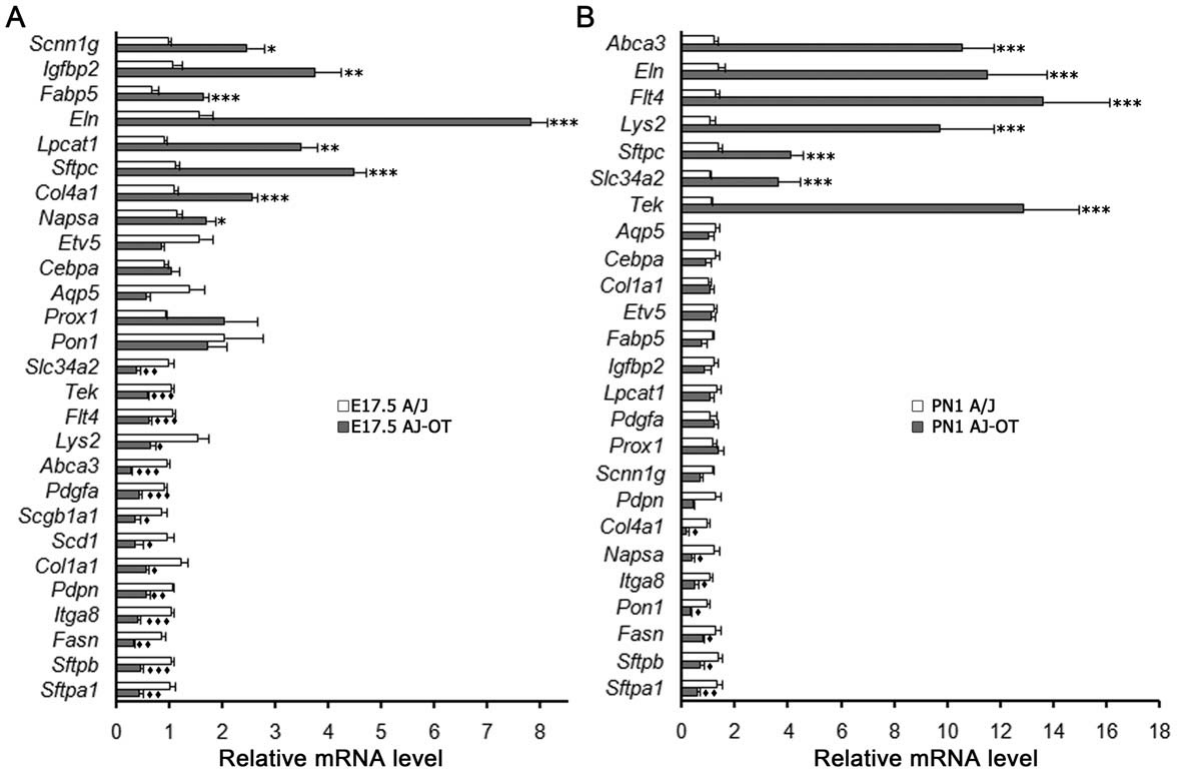
Both maternal environment and strain influenced mRNA expression associated with fetal and perinatal lung maturation. Quantitative RT-PCR for selected genes was performed to determine lung mRNA levels at E17.5 (A) and PN1 (B) from AJ-OT mice compared to mice from normal A/J dams. At E17.5, *Napsa*, *Eln*, *Fabp5*, *Scnn1g*, *Sftpc*, and *Lpcat1* mRNA levels were significantly induced in AJ-OT lungs. A subset of mRNAs, including *Abca3, Eln, Flt4, Lyz2, Sftpc, Slc34a2, and Tek*, was expressed at higher levels in the AJ-OT mice on PN1 compared to normal A/J mice. Results were normalized to *18S* rRNA and expressed as the means ± SEM of 12 animals per group; *p< 0.05, **p< 0.01, ***p< 0.001 for AJ-OT higher than A/J and ♦*p*< 0.05, ♦♦*p*< 0.01, ♦♦♦*p*< 0.001 for AJ-OT lower than A/J.

To determine whether the maternal environment or the strain of the fetus influenced gene expression associated with lung maturation, lung mRNAs from A/J mice born to A/J or C57BL/6J SCID dams were compared at E17.5 and PN1. At E17.5, expression of a subset of mRNAs was induced precociously in AJ-OT fetuses (Figure 5A). For example, *Napsa*, *Eln, Fabp5*, *Scnn1g*, *Sftpc*, and *Lpcat1* mRNAs, encoding proteins known to play important roles in surfactant lipid synthesis or perinatal lung maturation, were significantly induced in the AJ-OT mice, indicating that the maternal environment regulated these genes. Other subsets of mRNAs did not change and/or were reduced when comparing the AJ-OT with the A/J mice at E17.5 (Figure 5A). Likewise, a number of mRNAs were unchanged or reduced at PN1 in the AJ-OT mice when compared to those expressed in the A/J pups, indicating that these genes were regulated primarily by fetal genotype (Figure 5B). In contrast, a subset of genes associated with lung maturation, including *Sftpc*, *Abca3*, *Lys2*, *Flt4*, *Tek*, *Eln*, and *Scl34a2,* was expressed at higher levels in the AJ-OT pups at PN1 when compared to those expressed in the A/J pups (Figure 5B). Thus, maternal (C57BL/6J SCID) environment influenced the accelerated expression of subsets of genes associated with both prenatal and postnatal lung maturation in the AJ-OT fetuses and pups.

Analysis of the promoter regions of genes induced earlier in the AJ-OT fetuses at E17.5, identified a common regulatory cis-element, i.e., a binding site for HES/HEY-like transcription factor(s), known to be transcriptional effectors in the Notch pathway and to regulate cell differentiation and post-embryonic development [14], [15]. On the other hand, the promoters of genes induced in AJ-OT pups at PN1 were enriched in common regulatory cis-elements for C/EBPα and CREB (Tables S1 and S2). Our previous studies demonstrated that C/EBPα plays an important role in the maturation of the respiratory epithelium in late gestation. C/EBPα increases with advancing gestation and directly activates expression of a number of genes associated with pulmonary maturation. Its expression is required for the production of the surfactant lipids and proteins necessary for lung function at birth [13]. A recent study by Bird et al. demonstrated that Creb1 is required for differentiation of the respiratory epithelium during late developmental stages [16].

**Figure 6:**
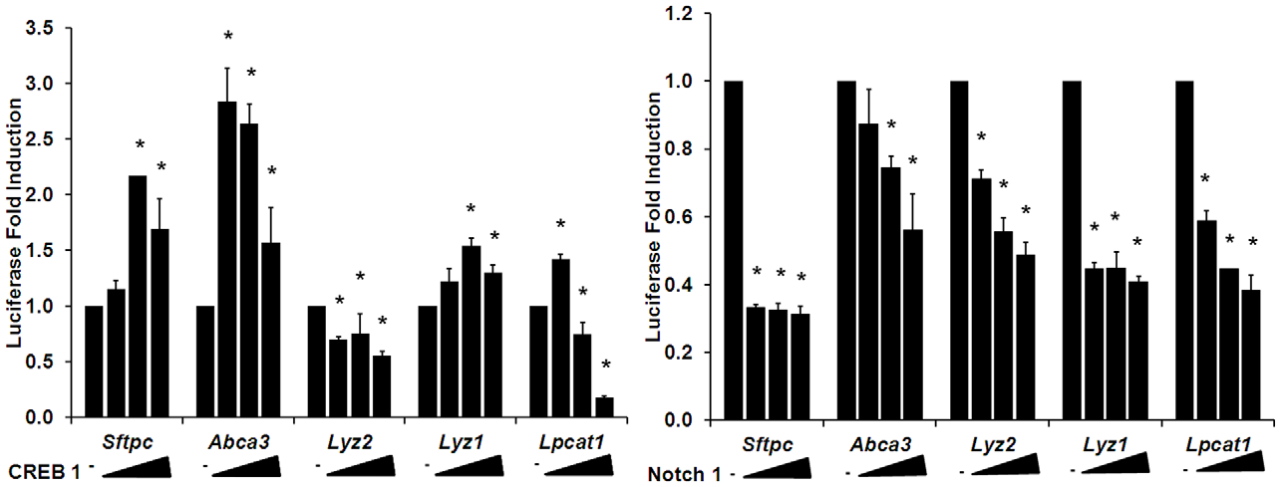
CREB1 and ΔE-Notch NICD regulate maturation associated genes *in vitro*. Dose response effects of CREB1 and activated Notch1 on mouse *Sftpc* (−4.8 kb), mouse *Abca3* (−2.6 kb), mouse *Lyz1* (−1.0 kb), mouse *Lyz2* (−1.0 kb), and mouse *Lpcat1* (−2.5 kb) promoter activities were assessed after cotransfection of promoter-luciferase constructs in Mouse Lung Epithelial (MLE)-15 cells *in vitro*. Luciferase activity was measured in the presence of increasing amounts of expression plasmids pCMV/CREB1 at 0, 0.05, 0.1, and 0.2 picomoles of DNA (A) and pCS2-ΔE-Notch NICD (activated form of Notch 1) at 0, 0.025, 0.05, and 0.1 picomoles of DNA (B). Results are expressed as the means ± SEM of 2 separate experiments performed in triplicate. *P<0.05 vs. control (pcDNA3 or pcDNA5 plasmid).

We compared lung mRNA expression profiles from *Creb* ^-/-^ mice at E17.5 (http://www.ebi.ac.uk/microarray-as/aer/; Accession number E-MEXP-1295) and *Cebpa* ^Δ/Δ^ gene targeted mice at E18 [13]. Many mRNAs important for lung function and maturation that were induced earlier in C57BL/6J than in A/J fetuses were similarly altered in lungs of *Cebpa* ^Δ/Δ^ and *Creb* ^-/-^ mice, including *Sftpa*, *Sftpb*, *Sftpd*, *Abca3*, *Scd1/2*, *Fabp5*, *Scnn1g*, and *Mia1*. Likewise, differentially expressed mRNAs in *Creb* ^-/-^ and *Cebpa* ^Δ/Δ^ mice were closely correlated (Table S2). While C/EBPα expression increases during prenatal lung maturation (Figure S1), we did not detect significant differences in levels of *Creb1* or *Creb3* mRNAs between the two strains (data not shown). The potential role of CREB1 was further assessed in transfection assays using promoters of several maturation-associated target genes *in vitro*, selecting genes that were induced in the AJ-OT pups at E17.5 and/or PN1 (Figure 6). CREB1 activated the expression of both *Abca3* and *Sftpc* but did not strongly influence *Lyz1*, *Lyz2* or *Lpcat1* promoter activity. *Lpcat1* expression was modestly induced at low concentrations of CREB plasmid but inhibited at higher doses. Consistent with the potential role of C/EBPα in the earlier induction of lung maturation markers, C/EBPα mRNA increased co-coordinately with *Abca3*, *Sftpb*, *Sftpc*, and *Lpcat1* mRNAs that increased earlier in the C57BL/6J strain with advancing gestation (Figure 3). The shared presence of potential CREB binding sites and the close correlation of lung mRNAs altered after deletion of CREB and C/EBPα support the concept that both C/EBPα and CREB serve important inductive roles in the timing of lung maturation.

Potential cis-regulatory elements predicting HES/HEY binding sites were identified in genes induced earlier in AJ-OT pups at E17.5, indicating potential regulation by the Notch pathway. Co-transfection with a plasmid expressing constitutively activated Notch repressed the expression of *Abca3*, *Sftpc*, *Lyz1*, *Lyz2*, and *Lpcat1* in promoter-reporter assays in mouse lung epithelial cells (Figure 6). Developmental changes in lung *Hes1* and *Hey1* mRNAs in the two mouse strains demonstrated that both *Hes1* and *Hey1* mRNAs were higher in A/J than in C57BL/6J pups at E17.5–18.5, suggesting a potential repressive role for Notch signaling in some aspects of perinatal lung maturation (Figure S7).

## Discussion

Gestational length, fetal growth, and maturation have been precisely balanced throughout evolution to ensure postnatal survival of placental mammals. While gestational length varies remarkably among mammals, parturition is precisely determined for each species. Organ maturation is also linked to gestational length and varies among species. In many mammals, including the human, differentiation of the fetal lung and production of pulmonary surfactant occurs primarily in the last trimester of gestation. Perinatal survival after preterm birth is limited by surfactant insufficiency causing RDS at birth. The present findings demonstrate that lung maturation was closely linked to gestational length in two different strains of mice. Ovarian transfer experiments demonstrated that biochemical aspects of fetal lung maturation were strongly influenced by the maternal genome to induce subsets of genes regulating perinatal lung function.

Lung maturation proceeded more rapidly in the C57BL/6J mice, as indicated by the earlier sacculation of peripheral airspaces and the expression of mRNAs regulating surfactant lipid and proteins. Likewise, the developmental increase in mRNAs associated with surfactant homeostasis, including those encoding proteins required for normal surfactant production and/or lipid homeostasis (such as, *Sftpc, Napsa, Fabp5,* and *Lpcat1*), occurred precociously in AJ-OT pups born early in C57BL/6J SCID dams. mRNAs induced precociously in the AJ-OT pups were associated with strain dependent differences in lung SatPC and lung weight observed during normal fetal lung development. While a number of genes regulating surfactant homeostasis were induced when AJ-OT pups were carried to term in the C57BL/6J SCID dams, many others were regulated in a strain-dependent manner and were not influenced by the maternal environment. Our studies demonstrate a remarkable, coordinate development of lung structure, as well as conducting and peripheral airway epithelial cell maturation, that coincides with the induction of biochemical and genetic markers of lung maturation that were regulated in a strain dependent manner. mRNAs regulating surfactant proteins and lipids, fluid homeostasis, and innate defense were induced with advancing gestation in a strain dependent manner.

The amiloride sensitive cation channel, SCNN1G, important for the clearance of lung liquid at birth, was also induced earlier in AJ-OT pups. *Scnn1g* gene targeted mice die of respiratory distress at birth caused by failure to clear fetal lung fluid [17], [18], a process that is also delayed in preterm infants [19]. Thus, the premature induction of *Scnn1g* in the AJ-OT pups born to C57BL/6J SCID dams provides a potential mechanism by which perinatal respiratory adaptation was enhanced.

Analysis of the promoter regions of genes induced earlier in the AJ-OT fetuses at E17.5, identified a common regulatory cis-element, a binding site for HES/HEY-like transcription factors (HELT), transcription factors known to regulate cell differentiation and post-embryonic development [14], [15] (Table S1A). In transfection assays, Notch inhibited expression of several maturation-associated lung mRNAs *in vitro*. In contrast, the transcription factors C/EBPα and CREB are required for perinatal lung maturation, and were shown in both the present and in previous studies to enhance expression of mRNAs associated with type II epithelial cell differentiation [13], [16]. The promoters of genes induced in AJ-OT pups at PN1 were enriched in common regulatory cis-elements for C/EBPα and CREB (Table S1B). While several important transcriptional modulators of lung maturation, such as *Nkx2.1* and *Foxa2*, did not change substantially during normal lung development, *Cebpa* mRNA increased in association with other aspects of lung maturation and was induced earlier in AJ-OT pups born to C57BL/6J SCID dams. In a previous study, deletion of *Cebpa* in the respiratory epithelium inhibited lung maturation and caused respiratory failure in newborn mice, indicating its critical role in perinatal survival [12], [13]. Likewise, a recent study demonstrated that *Creb1* ^-/-^ mice died from respiratory failure after birth [16]. In the present study, the role of Notch signaling in the maturational process was suggested but remains unclear. Analysis of the gene promoters associated with lung maturation predicted regulation by several HES/HEY DNA binding sites; however, *in vitro* promoter studies support a potential inhibitory role of Notch of several of the maturation-associated genes, including *Sftpc*, *Abca3*, and *Lpcat1*. The finding that both *Hes1* and *Hey1* mRNAs were increased in lung of A/J compared to C57BL/6J pups also supports a potential inhibitory role of the Notch pathway in the strain dependent differences in lung maturation observed in the present study (Figure S7).

Expression of the glucocorticoid receptor (*Nr3c1*) increased modestly with advancing gestation in both strains of mice. *Nr3c1* is required for normal lung maturation and perinatal survival, influencing the expression of a number of genes important for surfactant production [20]–[22]. While *Nr3c1* mRNA increased with advancing gestation, its levels were not clearly influenced by maternal strain.

The present finding, that maternal genes influence lung maturation, suggests a mechanism by which survival of preterm infants can be salvaged, i.e., by which the timing of parturition and lung maturation can be adjusted to enhance perinatal survival. Expression of a subset of genes regulating surfactant homeostasis and lung fluid clearance increased in a process linking gestational age and lung maturation during the prenatal period of development. In ovarian transfer experiments, timing of parturition was determined by the maternal strain and was independent of fetal or paternal genetic background. Both lung growth and expression of a subset of genes regulating perinatal lung maturation and function were influenced by the genotype of the dam. The present findings demonstrate a correlation between maternal strain, gestational length, and lung maturation, a correlation that is also supported by genetic evidence in humans regarding the importance of maternal, rather than paternal, inheritance of risk for preterm birth [23], [24]. The present work provides evidence of a complex transcriptional network by which the maternal environment/genome influences lung maturation and perinatal survival.

## Materials and Methods

### Animal husbandry and ovary transfer

C57BL/6J and A/J mice between 6 and 8 weeks of age were housed in humidity- and temperature-controlled rooms on a 12-hour light:dark cycle at The Jackson Laboratory (Bar Harbor, ME). Mice were maintained in a pathogen-free environment in accordance with protocols approved by the Jackson Laboratory Animal Care and Use Committee. Mice were allowed food and water *ad libitum*. Pair and trio mating were set at the end of the day. The dams were approximately 9–10 weeks at the time of mating. A vaginal plug found the following morning indicated successful mating. Fertilization (0 hour) was calculated at the midpoint of the dark cycle, prior to the appearance of the plug. Females were monitored continuously from E14.5 using infrared lighting and closed circuit, infrared-video cameras (Inter-Pacific, Wheeling, IL). Video recordings identified the birth of the first pup to assign gestational length [9]. The interval between all time points was 24 hours, except for E20.5 (just prior to birth) and P0 (within 3–6 hours post birth) for the A/J strain.

Transfer of A/J ovaries (albino) to C57BL/6J-SCID (black) mice was performed as described previously [9], [25]. Briefly, females (both donors and receptors) used for ovarian transfer were 4–5 weeks old. Females were then mated for their first litters (non-timed mating) immediately after the 1-week post-op healing period. After successful ovarian transfer, timed matings of C57BL/6J-SCID females (with C57BL/6J ovaries) with C57BL/6J males, and C57BL/6J-SCID females (with A/J ovaries) with A/J males were set up to determine differences in gestational length. Success of the ovarian transplants was determined by delivery of albino litters and by genotyping. For consistency, only male fetuses, pups and adults were used for the morphological, biochemical, and mRNA data collected for these studies.

### Respiration after preterm birth

Pregnant C57BL/6J and A/J dams were euthanized at E18.5 or E19.5 (A/J only) and fetuses were removed from the uterus and amniotic membranes. Resuscitation of the pups was performed by clearing nostrils and mouth and gently massaging the pups until they moved and breathed on their own. Pups were considered dead if attempts to resuscitate pups failed after 20 minutes. After resuscitation, pups were kept on a warming pad and observed for at least 2 hrs.

### Tissue, mRNA and lipid analyses

Lung tissue preparation, immunohistochemistry, morphometry, RNA and lipid extraction were performed as described [13], [26], [27] and in Data S1.

### Cell culture and promoter reporter assays

Mouse lung epithelial (MLE-15) cells were maintained as previously described [28]. Cells were plated at 2×10^5^ per well in 6-well plate and transfected 24 hours later with Fugene 6 (Roche, Indianapolis, IN) according to manufacturer's instructions. Effects of CREB1 and Notch1 were assessed in promoter assays tested on the mouse *Sftpc* (−4.8 kb), mouse *Abca3* (−2.6 kb), mouse *Lyz1* (−1.0 kb), mouse *Lyz2* (−1.0 kb) and mouse *Lpcat1* (−2.5 kb) genes with either pGL2 or pGL3 as the backbone vector (Promega, Madison, WI). Methods are provided in Data S1.

## Supporting Information

Figure S1 **Delayed expression of C/EBPα in the A/J fetuses during the perinatal period.** Immunostaining for C/EBPα was performed on C57BL/6J and A/J lungs from fetal mice harvested at E15.5, 16.5, E17.5, E18.5, and E19.5 (A/J only). At E15.5, C/EBPα was detected in both epithelial (arrows) and mesenchymal cells (arrowheads). At E16.5, C/EBPα was increasingly restricted to the epithelium in C57BL/6J mice, while still present in both tissue compartments in the A/J mice. During the saccular stage, C/EBPα increased in both strains. At E18.5, C/EBPα was increased in alveolar regions and was detectable in the conducting airway epithelium (Br). While C/EBPα expression was present in most epithelial cells lining the distal conducting airways (asterisk) of C57BL/6J mice on E18.5 (G), fewer epithelial cells expressed C/EBPα in A/J lungs (H). By E19.5, C/EBPα expression in A/J fetal lungs was similar to that in C57BL/6J fetuses at E18.5 (I). Scale bar: 100 µm.
(TIFF)

Figure S2 **FOXA2 is similar in C57BL/6J and A/J.** Immunostaining for FOXA2, a transcription factor expressed in lung epithelial cells, was performed on C57BL/6J and A/J lungs from fetal mice harvested at E15.5, 16.5, E17.5, E18.5, and E19.5 (A/J only). Ontogenic changes in FOXA2 staining were similar in respiratory epithelial cells of both mouse strains. Scale bar: 100 µm.
(TIFF)

Figure S3 **Earlier expression of CCSP in conducting C57BL/6J mice.** Staining for CCSP (a Clara cell marker), was performed on C57BL/6J and A/J fetal mouse lungs harvested at E15.5, 16.5, E17.5, E18.5, and E19.5 (A/J only). At E17.5 and E18.5, CCSP immunostaining was increased in bronchiolar epithelial cells in the C57BL/6J compared to A/J fetuses. By E19.5, CCSP staining in A/J was similar to that seen at E18.5 C57BL/6J fetuses. Scale bar: 30 µm.
(TIFF)

Figure S4 **Earlier expression of acetylated tubulin in conducting airways of lungs from C57BL/6J than in A/J mice.** Immunohistochemical staining for acetylated tubulin (a ciliated cell marker), was performed on C57BL/6J and A/J lungs from fetal mice harvested at E15.5, 16.5, E17.5, E18.5, and E19.5 (A/J only). α-Tubulin was detected as early as E16.5 in the bronchiolar epithelium of C57BL/6J fetuses. α-Tubulin was first detected at E18.5 in A/J mice. α-Tubulin was similar in E19.5 A/J and E18.5 C57BL/6J mice. Scale bar: 20 µm.
(TIFF)

Figure S5 **mRNA expression profiles during lung development.** Dynamic mRNA expression profiles of 53 genes at different gestation ages for the A/J mice were correlated with body weight (BW), lung weight (LW), SatPC (µmol/gLW, µmol/gBW, and total), and morphometric measurements (airspace, tissue) at corresponding gestational ages using multivariate correction function from JMP 9 (SAS Institute Inc, NC). The heat map was generated based on data from A/J mice using Ward's minimum variance method to estimate cluster similarity. Gradients in the red and green color range indicate positive and negative correlation, respectively. The levels of mRNAs in red clusters were highly correlated with ontogenic changes in lung SatPC and fractional area of airspace; mRNAs in blue clusters were moderately correlated with SatPC, but closely correlated with fractional area of airspace; mRNAs in green clusters correlated well with the fractional area of the tissue compartment.
(TIFF)

Figure S6 **Prenatal and postnatal growth after ovarian transfer.** Body weight (A), lung weight (B), and lung-to-body weight ratios (C) of OT-B6 and OT-AJ mice were measured at E17.5 and at PN1. Values are means ± SEM of 12 animals per strain derived from n = 6 dams; *p< 0.05, for OT-AJ vs. OT-B6. Lung weights and LW/BW ratios were increased when A/J fetuses were born to C57BL/6J dams. SatPC was measured in lungs from B6-OT and AJ-OT mice at E17.5 and PN1 (D-E). Results are expressed as the means ± SEM of 12 animals per strain.
(TIFF)

Figure S7 **Hey1 and Hes1 mRNAs were compared in whole lung mRNA from C57BL/6J and A/J pups at each age.** Statistical differences were assessed by ANOVA, * indicate p<0.05.
(TIFF)

Table S1 **Common cis-elements in the promoter regions of genes induced in AJ-OT mice at E17.5 and at PN1.**
(DOCX)

Table S2 **Differentially expressed mRNAs in** ***Creb*** **^-/-^ and** ***Cebpa*** ^Δ**/**Δ^ **mice.**
(DOCX)

Data S1 **Online Data Supplement – Materials and Methods.**
(DOCX)
