# Supplementary figures and images for: Maternal Synchronization of Gestational Length and Lung Maturation
Source: PLoS One. 2011 Nov 9;6(11):e26682. doi: 10.1371/journal.pone.0026682 (PMC3212521; doi:10.1371/journal.pone.0026682)

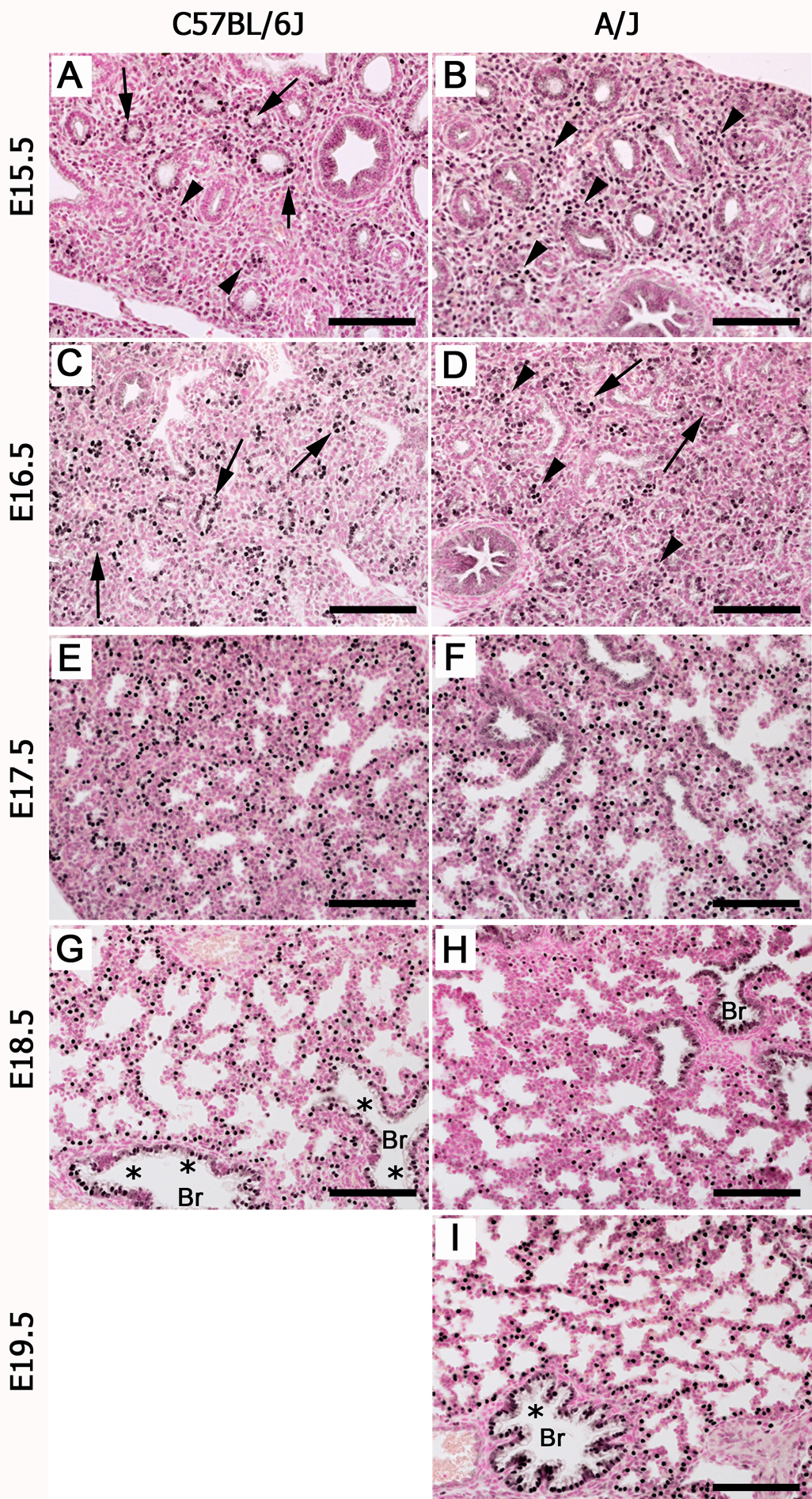

Supplement: Figure S1 — Delayed expression of C/EBPα in the A/J fetuses during the perinatal period. Immunostaining for C/EBPα was performed on C57BL/6J and A/J lungs from fetal mice harvested at E15.5, 16.5, E17.5, E18.5, and E19.5 (A/J only). At E15.5, C/EBPα was detected in both epithelial (arrows) and mesenchymal cells (arrowheads). At E16.5, C/EBPα was increasingly restricted to the epithelium in C57BL/6J mice, while still present in both tissue compartments in the A/J mice. During the saccular stage, C/EBPα increased in both strains. At E18.5, C/EBPα was increased in alveolar regions and was detectable in the conducting airway epithelium (Br). While C/EBPα expression was present in most epithelial cells lining the distal conducting airways (asterisk) of C57BL/6J mice on E18.5 (G), fewer epithelial cells expressed C/EBPα in A/J lungs (H). By E19.5, C/EBPα expression in A/J fetal lungs was similar to that in C57BL/6J fetuses at E18.5 (I). Scale bar: 100 µm. (TIFF) [file pone.0026682.s001.tiff]

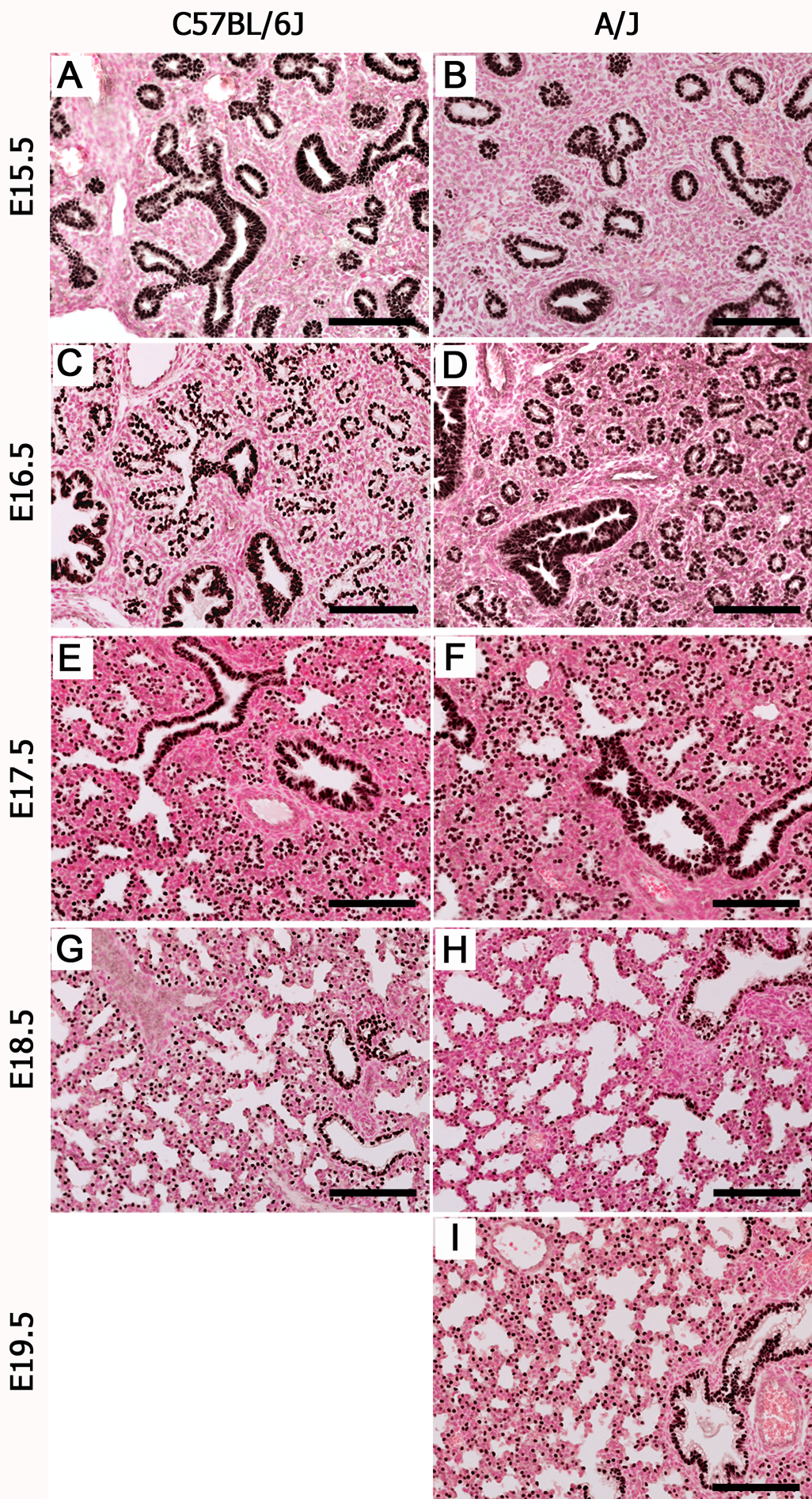

Supplement: Figure S2 — FOXA2 is similar in C57BL/6J and A/J. Immunostaining for FOXA2, a transcription factor expressed in lung epithelial cells, was performed on C57BL/6J and A/J lungs from fetal mice harvested at E15.5, 16.5, E17.5, E18.5, and E19.5 (A/J only). Ontogenic changes in FOXA2 staining were similar in respiratory epithelial cells of both mouse strains. Scale bar: 100 µm. (TIFF) [file pone.0026682.s002.tiff]

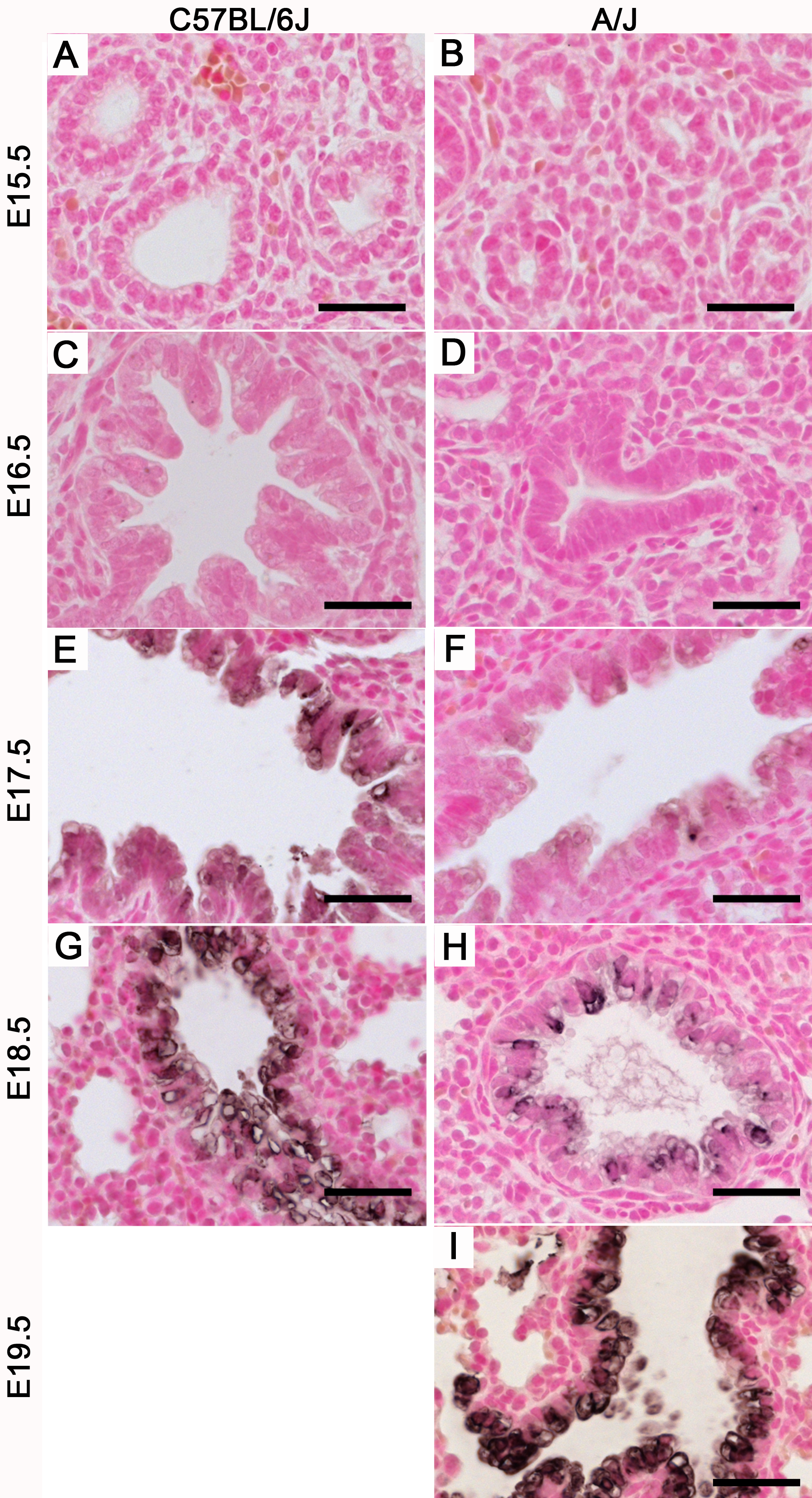

Supplement: Figure S3 — Earlier expression of CCSP in conducting C57BL/6J mice. Staining for CCSP (a Clara cell marker), was performed on C57BL/6J and A/J fetal mouse lungs harvested at E15.5, 16.5, E17.5, E18.5, and E19.5 (A/J only). At E17.5 and E18.5, CCSP immunostaining was increased in bronchiolar epithelial cells in the C57BL/6J compared to A/J fetuses. By E19.5, CCSP staining in A/J was similar to that seen at E18.5 C57BL/6J fetuses. Scale bar: 30 µm. (TIFF) [file pone.0026682.s003.tiff]

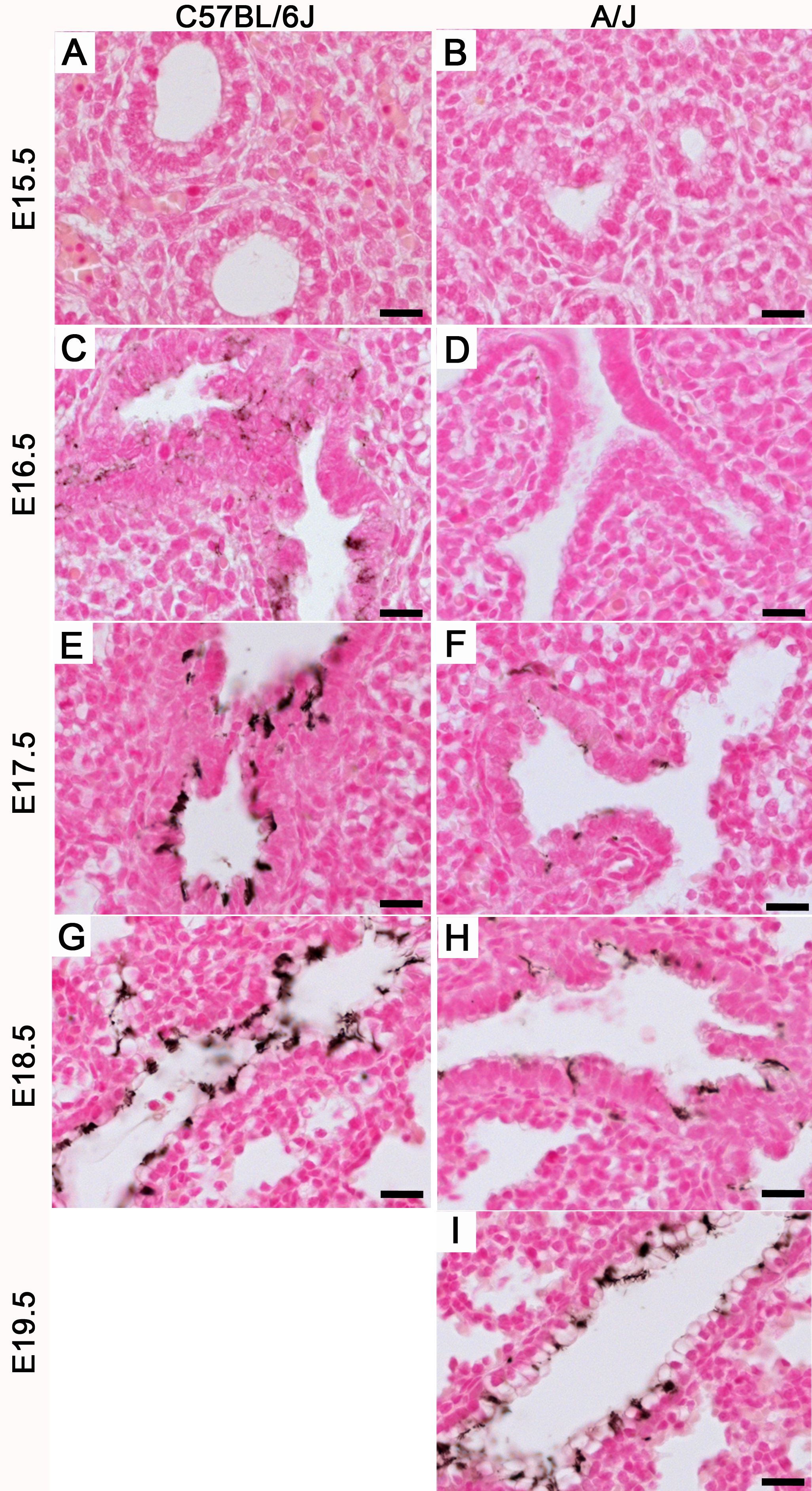

Supplement: Figure S4 — Earlier expression of acetylated tubulin in conducting airways of lungs from C57BL/6J than in A/J mice. Immunohistochemical staining for acetylated tubulin (a ciliated cell marker), was performed on C57BL/6J and A/J lungs from fetal mice harvested at E15.5, 16.5, E17.5, E18.5, and E19.5 (A/J only). α-Tubulin was detected as early as E16.5 in the bronchiolar epithelium of C57BL/6J fetuses. α-Tubulin was first detected at E18.5 in A/J mice. α-Tubulin was similar in E19.5 A/J and E18.5 C57BL/6J mice. Scale bar: 20 µm. (TIFF) [file pone.0026682.s004.tiff]

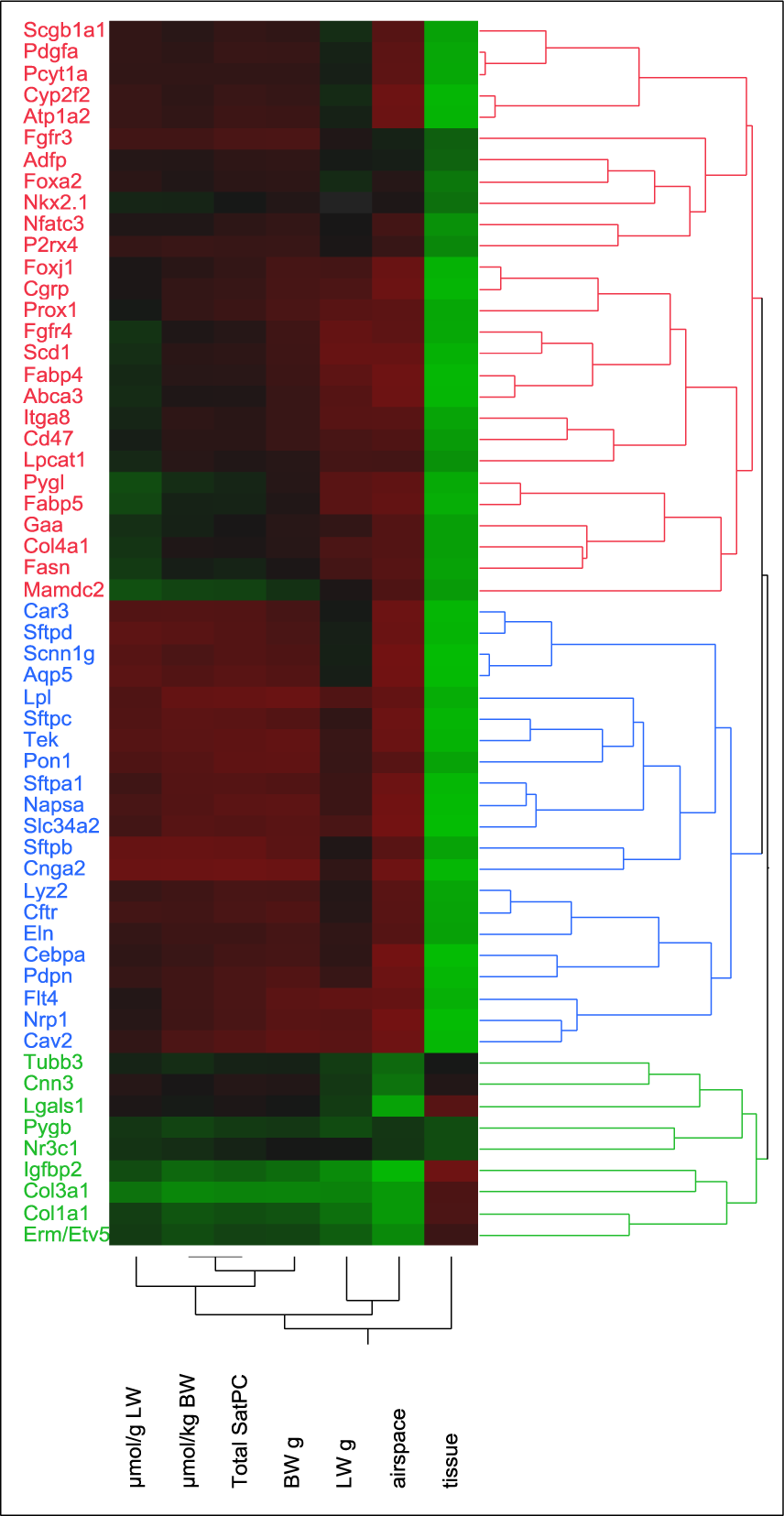

Supplement: Figure S5 — mRNA expression profiles during lung development. Dynamic mRNA expression profiles of 53 genes at different gestation ages for the A/J mice were correlated with body weight (BW), lung weight (LW), SatPC (µmol/gLW, µmol/gBW, and total), and morphometric measurements (airspace, tissue) at corresponding gestational ages using multivariate correction function from JMP 9 (SAS Institute Inc, NC). The heat map was generated based on data from A/J mice using Ward's minimum variance method to estimate cluster similarity. Gradients in the red and green color range indicate positive and negative correlation, respectively. The levels of mRNAs in red clusters were highly correlated with ontogenic changes in lung SatPC and fractional area of airspace; mRNAs in blue clusters were moderately correlated with SatPC, but closely correlated with fractional area of airspace; mRNAs in green clusters correlated well with the fractional area of the tissue compartment. (TIFF) [file pone.0026682.s005.tiff]

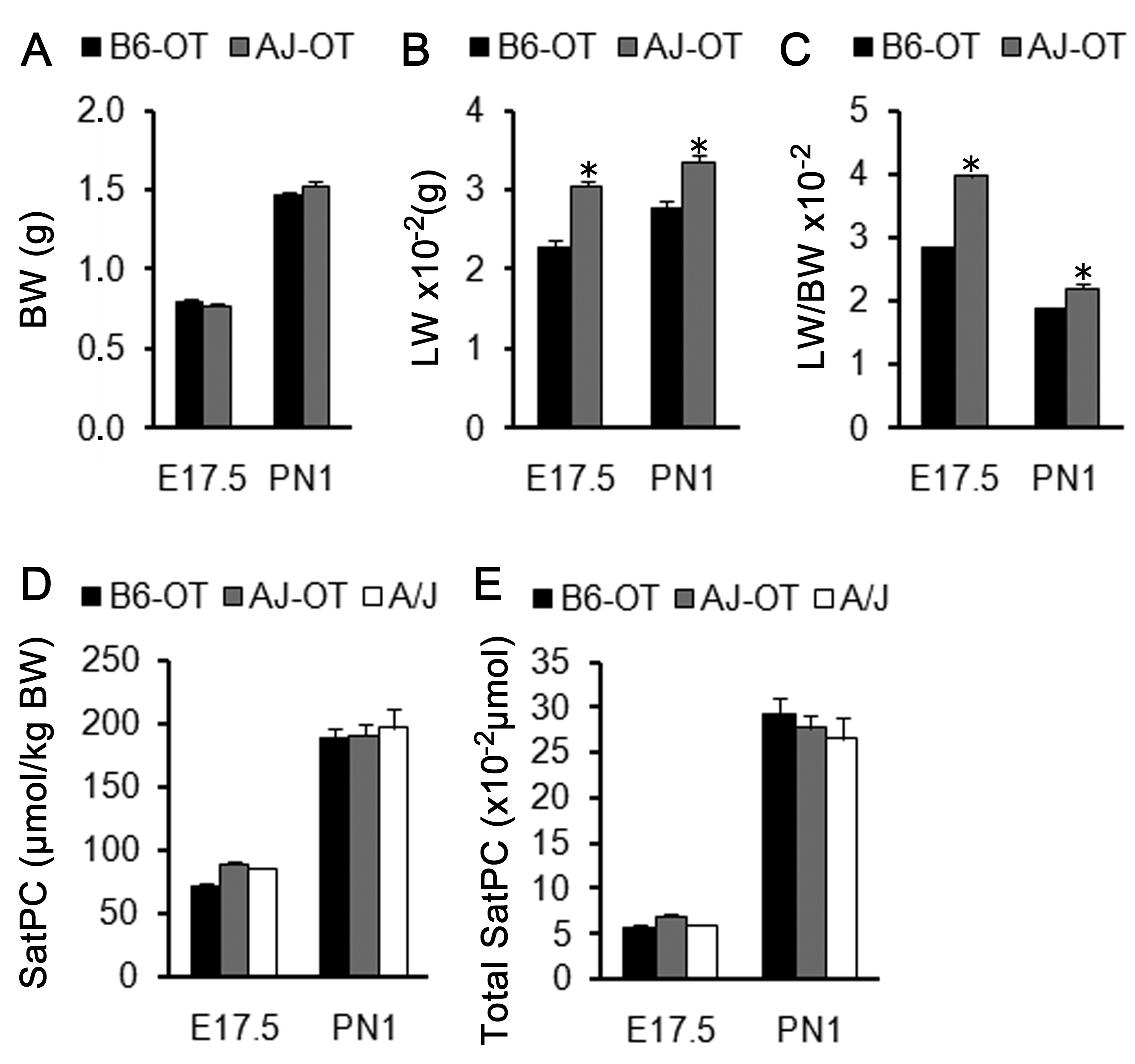

Supplement: Figure S6 — Prenatal and postnatal growth after ovarian transfer. Body weight (A), lung weight (B), and lung-to-body weight ratios (C) of OT-B6 and OT-AJ mice were measured at E17.5 and at PN1. Values are means ± SEM of 12 animals per strain derived from n = 6 dams; *p< 0.05, for OT-AJ vs. OT-B6. Lung weights and LW/BW ratios were increased when A/J fetuses were born to C57BL/6J dams. SatPC was measured in lungs from B6-OT and AJ-OT mice at E17.5 and PN1 (D-E). Results are expressed as the means ± SEM of 12 animals per strain. (TIFF) [file pone.0026682.s006.tiff]

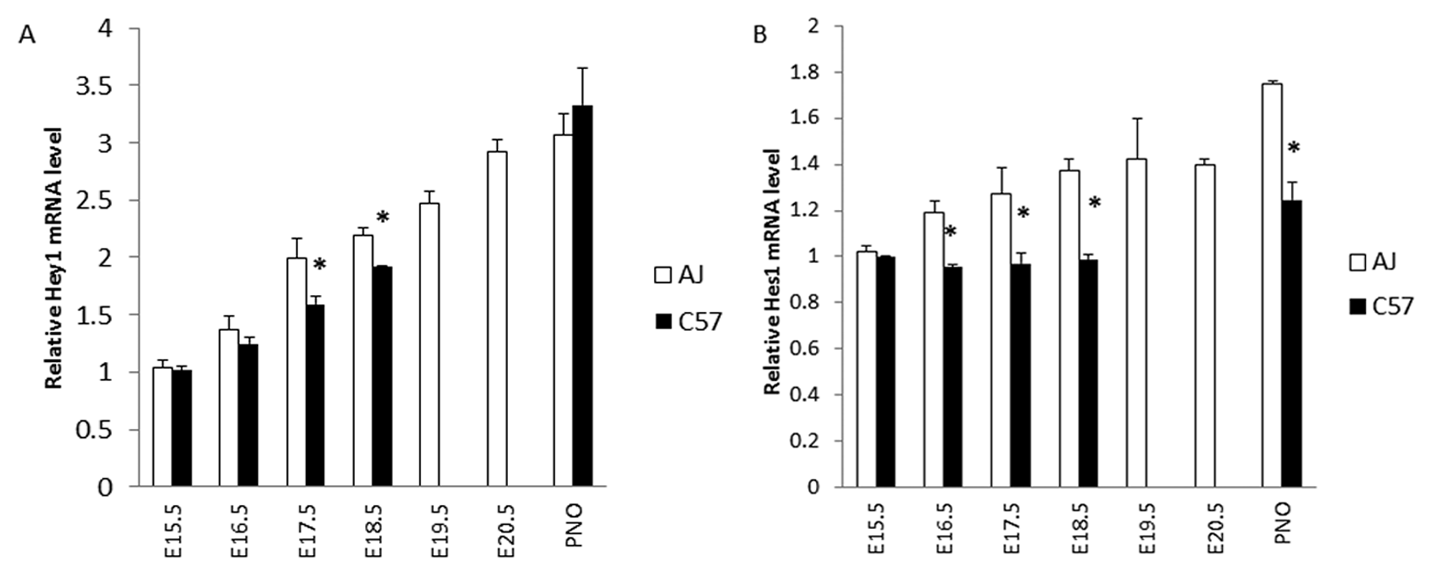

Supplement: Figure S7 — Hey1 and Hes1 mRNAs were compared in whole lung mRNA from C57BL/6J and A/J pups at each age. Statistical differences were assessed by ANOVA, * indicate p<0.05. (TIFF) [file pone.0026682.s007.tiff]
