# Supplementary material for: Maternal Synchronization of Gestational Length and Lung Maturation
Source: PLoS One. 2011 Nov 9;6(11):e26682. doi: 10.1371/journal.pone.0026682 (PMC3212521; doi:10.1371/journal.pone.0026682)
Supplement: Table S1 — Common cis-elements in the promoter regions of genes induced in AJ-OT mice at E17.5 and at PN1. (DOCX) [file pone.0026682.s008.docx]

Table S1a. **Common cis-elements in the promoter regions of genes induced in AJ-OT mice at E17.5.**

| **Family/Matrix** | **p-value** | **Lpcat1** | **Eln** | **Sftpc** | **Napsa** | **Scnn1g** | **Fabp5** | **Col4a1** | **Igfbp2** |
| --- | --- | --- | --- | --- | --- | --- | --- | --- | --- |
| **V$HESF/V$HELT.01** | **1.62E-06** | **2** | **1** | **2** | **1** | **1** | **1** | **1** | **3** |
| V$YBXF/V$YB1.01 | 2.90E-04 | 0 | 1 | 1 | 1 | 1 | 1 | 1 | 1 |
| V$EGRF/V$EGR1.01 | 4.02E-04 | 1 | 1 | 1 | 0 | 1 | 1 | 2 | 5 |
| V$NR2F/V$HNF4.01 | 7.58E-04 | 2 | 2 | 1 | 2 | 1 | 0 | 2 | 3 |
| V$NR2F/V$HPF1.01 | 8.96E-04 | 5 | 3 | 1 | 2 | 1 | 0 | 1 | 2 |
| V$HNF1/V$HNF1.03 | 3.13E-03 | 1 | 1 | 0 | 2 | 1 | 2 | 1 | 2 |
| V$HNF1/V$HNF1.01 | 3.44E-03 | 1 | 1 | 0 | 2 | 1 | 1 | 1 | 1 |
| V$HOXF/V$HOXB8.01 | 3.44E-03 | 1 | 2 | 1 | 1 | 2 | 1 | 1 | 0 |
| V$PERO/V$PPARG.03 | 4.07E-03 | 1 | 3 | 1 | 3 | 1 | 1 | 0 | 1 |
| V$SP1F/V$SP1.01 | 4.22E-03 | 1 | 12 | 1 | 0 | 1 | 3 | 2 | 13 |

Table S1b. **Common cis-elements in the promoter regions of genes induced in AJ-OT mice at PN1**

| **Family/Matrix** | **p-value** | **Abca3** | **Eln** | **Flt4** | **Lyz2** | **Sftpc** | **Slc34a2** | **Tek** |
| --- | --- | --- | --- | --- | --- | --- | --- | --- |
| **V$CREB/V$E4BP4.01** | **9.37E-04** | **1** | **2** | **1** | **3** | **1** | **2** | **1** |
| **V$CEBP/V$CEBP.02** | **1.06E-03** | **2** | **2** | **1** | **1** | **1** | **3** | **2** |
| V$TEAF/V$TEAD.01 | 3.86E-03 | 1 | 0 | 1 | 4 | 1 | 1 | 1 |
| V$MYBL/V$CMYB.01 | 3.86E-03 | 4 | 0 | 1 | 1 | 1 | 1 | 1 |
| V$TEAF/V$TEF1.01 | 4.09E-03 | 1 | 0 | 1 | 4 | 1 | 1 | 1 |
| V$SORY/V$SOX9.03 | 4.09E-03 | 2 | 2 | 1 | 2 | 1 | 2 | 0 |
| V$HAML/V$AML3.01 | 4.32E-03 | 1 | 0 | 3 | 2 | 1 | 1 | 3 |
| V$PARF/V$TEF_HLF.01 | 8.45E-03 | 0 | 3 | 1 | 1 | 1 | 3 | 1 |
| V$MAZF/V$MAZ.01 | 1.12E-02 | 3 | 2 | 3 | 0 | 1 | 2 | 1 |
| V$PARF/V$TEF.01 | 2.19E-02 | 0 | 2 | 1 | 4 | 1 | 1 | 4 |

Note: Using MatInspector (Genomatix Software GmbH), we searched for common cis-elements in the promoter region (-1000bp to +200bp) of genes for which expression was induced in AJ-OT at E17.5 (E1a) and at PN1 (E1b). The transcription binding site is determined based on the Genomatix weighted Matrix Library 8.3. Matrix families are groups of weight matrices for the same or functionally similar transcription factors. V$ stands for vertebrates.
