# Supplementary material for: Maternal Synchronization of Gestational Length and Lung Maturation
Source: PLoS One. 2011 Nov 9;6(11):e26682. doi: 10.1371/journal.pone.0026682 (PMC3212521; doi:10.1371/journal.pone.0026682)
Supplement: Table S2 — Differentially expressed mRNAs in Creb -/- and Cebpa Δ/Δ mice. (DOCX) [file pone.0026682.s009.docx]

Table S2

| **Gene** | **Fold change(Creb1^-/-^)** | **Fold change(Cebpa^Δ/Δ^)** |
| --- | --- | --- |
| **Scd1** | -5.08 | -21.1 |
| **Slc34a2** | -2.49 | -7.5 |
| **Eln** | -1.73 | -1.43 |
| **Abca3** | -1.53 | -3.38 |
| **Scd2** | -5.08 | -2.53 |
| **Sftpc** | -1.20 | -1.2 |
| **Flt4** | -1.20 | -1.2 |
| **Tek** | NC | NC |

NC: not changed. T-Test P-value > 0.05.
