# Supplementary material for: Maternal Synchronization of Gestational Length and Lung Maturation
Source: PLoS One. 2011 Nov 9;6(11):e26682. doi: 10.1371/journal.pone.0026682 (PMC3212521; doi:10.1371/journal.pone.0026682)
Supplement: Data S1 — Online Data Supplement – Materials and Methods. (DOCX) [file pone.0026682.s010.docx]

**ONLINE DATA SUPPLEMENT**

**Maternal Synchronization of Gestational Length and Lung Maturation**

Valérie Besnard

Susan E. Wert

Machiko Ikegami

Yan Xu

Caleb Heffner

Stephen A. Murray

Leah Rae Donahue

Jeffrey A. Whitsett

**Material and Methods**

**Lung RNA extraction and quantitative RT-PCR.** Lungs from 4 litters (3 males/litter) of each strain were divided in half and homogenized for either RNA (right lung) or lipid analysis (left lung) at each gestational and postnatal time point. The interval between all time points was 24 hours, except for E20.5 (just prior to birth) and P0 (just after birth) for the A/J strain, for which the perinatal interval was 3 to 6 hours. RNA was isolated from whole right lung homogenates using an RNAeasy Protect mini kit (Qiagen, Valencia, CA). Samples were pooled for each litter, and quantitative PCR using 53 Taqman probes (Applied BioSystems, Foster City, CA) was performed with the primer sets listed. Probe and primer sets for *18s* rRNA were used for normalization. PCR reactions and relative quantification were performed using 25 ng of cDNA per reaction in a StepOnePlus Real-Time PCR system (Applied Biosystems).

**Lung RNA extraction, RT-PCR analysis.** Quantitative PCRs using Taqman probes were performed with the following primer sets specific for: *Abca3* (Mm00550501_m1), *Lpl* (Mm00434770_m1), *Adfp* (Mm00475794_m1), *Lyz2* (Mm01612741_m1), *Aqp5* (Mm00437578_m1), *Mamdc2* (Mm00805078_m1), *Atp1a2* (Mm00617899_m1), *Napsa* (Mm00492829_m1), *Calca* (Mm00801463_g1), *Nfatc3* (Mm01249200_m1), *Car3* (Mm00483016_m1), *Nkx2.1* (Mm00447558_m1), *Cav2* (Mm00516827_m1), *Nr3c1* (Mm00433832_m1), *Cd47* (Mm00495005_m1), *Nrp1* (Mm00435372_m1), *Cdipt* (Mm00513684_m1), *Pcyt1a* (Mm00447774_m1), *Cebpa* (Mm01265914_s1), *Pdgfa* (Mm00435540_m1), *Cftr* (Mm00445197_m1), *Pdpn1* (Mm00494716_m1), *Cnga2* (Mm00432614_m1), *Pon1* (Mm00599936_m1), *Cyp2f2* (Mm00484087_m1), *Pr2x4* (Mm00501787_m1), *Eln* (Mm00514670_m1), *Prox1* (Mm00435969_m1), *Etv5* (Mm00465816_m1), *Pygb* (Mm00464080_m1), *Fabp4* (Mm00445880_m1), *Pygl* (Mm00500078_m1), *Fabp5* (Mm00783731_s1), *Scd1* (Mm01197142_m1), *Fasn* (Mm01253292_m1), *Scgb1A1* (Mm00442046_m1), *Fgfr3* (Mm00433294_m1), *Scnn1g* (Mm00441228_m1), *Fgfr4* (Mm00433314_m1), *Sftpa1* (Mm00499170_m1), *Flt4* (Mm00433337_m1), *Sftpb* (Mm00455681_m1), *Foxa2* (Mm01976556_s1), *Sftpc* (Mm00488144_m1), *Foxj1* (Mm00807215_m1), *Sftpd* (Mm00486060_m1), *Gaa* (Mm00484581_m1), *Slc34a2* (Mm01215846_m1), *Itga8* (Mm01324958_m1), *Tek* (Mm00443254_m1), *Lpcat1* (Mm00461015_m1), *Tubb3* (Mm00727586_s1).

**Tissue preparation, histology, and immunohistochemistry. For each developmental time point, 3 to 4 pups from at least 4 different litters were fixed in 4% paraformaldehyde, rinsed in phosphate-buffered saline, dehydrated through a graded series of ethanol solutions to xylene, embedded in paraffin, and sectioned for analysis. Body weights were determined at time of harvest and found to be statistically identical to those mice harvested for biochemical and molecular analysis. I**mmunohistochemistry was performed on 5-micron sections using an immunoperoxidase method (VECTASTAIN Elite ABC kit, Vector Laboratories, Burlingame CA), followed by counterstaining with 0.1% nuclear fast red. Primary antibodies were used at the following dilutions: pan-cytokeratin (1:500, mouse IgG1, Sigma, St Louis, MO), LPCAT1 (1:500, guinea pig polyclonal) [1], mature SP-B (1:500, rabbit polyclonal, Seven Hills Bioreagents, Cincinnati, OH), pro-SP-C (1:500, rabbit polyclonal, Seven Hills Bioreagents, Cincinnati, OH), C/EBPα (1:2,000, rabbit polyclonal, Santa Cruz Biotechnology Inc., Santa Cruz, CA), FOXA2 (1:4,000, rabbit polyclonal, Seven Hills Bioreagents), acetylated α-tubulin (Clone6-11B-1, IgG2b, 1:2000, Sigma, St Louis, MO), Scgb1a1 (goat polyclonal, 1:5000, Santa Cruz Biotechnology Inc.). Biotinylated secondary antibodies directed to primary antibody host IgGs were used at 1:200 (Vector Laboratories, Burlingame CA). All experiments shown are representative of findings from at least 4 to 12 mice, selected from four different litters, with body weights clustered around the mean for each strain.

**Morphometry.** Both growth velocity and morphometric measurements were established for fetal lungs harvested on E15.5, E16.5, E17.5, and E18.5 for both strains, and on E19.5 for the A/J strain, since **C57BL/6J dams delivered at E19.5 and A/J dams delivered on E20.5** (n=4 animals for each strain and time point). The relative proportion or percent fractional area (% FX area) of both air space and the tissue compartment was determined by a point-counting method using MetaMorph imaging software (Molecular Devices, Sunnyvale, CA) [2, 3]. Four mice of both strains (selected from four different litters as described above) were studied at each developmental time point. Measurements were performed on one section containing both left and right lungs. Ten randomly selected fields per section were analyzed to gather the data. The x- and y-coordinates for each field measured were selected by a random number generator. A computer-generated, 108-point lattice grid was superimposed on each field, and the number of intersections (points) falling over air space or tissue was counted.

**Lipid Analysis**. SatPC was analyzed in pooled homogenates of the left lung from 3 mice at each time point (n=4 pools) [4]. Lung homogenates were extracted with chloroform:methanol (2:1), and SatPC was isolated from the lipid extract after osmium tetroxide oxidation and quantified by measuring inorganic phosphorus [5].

**Cell culture, transfection and promoter reporter assays**. Six-well plates were treated with 0.1 pM promoter-luciferase reporter plasmid per well and when needed 0.025, 0.05, 0.1, or 0.2 pM of specific expression plasmid pCMV CREB1 or pCS2-ΔE-Notch NICD (activated form of Notch1). The amount of transfected plasmid was kept constant by the addition of corresponding backbone DNA, either pcDNA3 or pcDNA5. pCMV-ß-galactosidase at 0.02 pM was also transfected as the internal control. After approximately 24 hours, lysates were collected and assayed for ß-galactosidase and luciferase activities (Promega, Madison, WI). Light units were measured by luminometry (AutoLumat Plus LB 953, Berthold Technologies, Oak Ridge, TN). Firefly luciferase activities were normalized (in relative light units) to ß-galactosidase activity. All assays were performed in triplicate in at least 2 separate experiments.

**Statistical analysis**. Differences between groups were analyzed by applying two-way ANOVA. **Tukey**'s *post hoc* test was used to compare times and groups when there was statistical significance. Values were expressed as mean ± SEM, and *p values* less than *0.05* were considered statistically significant. The heat map and clustering of differentially expressed mRNAs was generated using Ward’s minimum variance methods [6,7].

**mRNA analysis.** Data were normalized to mRNA levels at E15.5 and Log2 transformed for each strain. Normalized data were subjected to hierarchical clustering using complete linkage to calculate distance between clusters. mRNAs were correlated with total saturated phosphatidylcholine (SatPC), body weight, lung weight, and normalized SatPC (relative to lung weight and body weight) by Pearson product-moment correlation coefficient. Lung mRNA expression from E17.5 *Creb*^-/-^ mice and *Cebpa*^∆/∆^ gene targeted mice at E18 were obtained from http://www.ebi.ac.uk/microarray-as/aer/, Accession number E-MEXP-1295, and [8] respectively.

**References**

1. Bridges JP, Ikegami M, Brilli LL, Chen X, Mason RJ, et al. (2010) LPCAT1 regulates surfactant phospholipid synthesis and is required for transitioning to air breathing in mice. J Clin Invest 120: 1736-1748.

2. Besnard V, Wert SE, Kaestner KH, Whitsett JA. (2005) Stage-specific regulation of respiratory epithelial cell differentiation by Foxa1. Am J Physiol 289: L750-L759.

3. Bolender RP, Hyde DM, Dehoff RT. (1993) Lung morphometry: a new generation of tools and experiments for organ, tissue, cell, and molecular biology. Am J Physiol 265: L521-L548.

4. Bartlett GR. (1959) Phosphorus assay in column chromatography. J Biol Chem 234: 466-468.

5. Jobe A, Kirkpatrick E, Gluck L. (1978) Labeling of phospholipids in the surfactant and subcellular fractions of rabbit lung. J Biol Chem 253: 3810-3816.

6. Milligan GW. (1980) An Examination of the Effect of Six Types of Error Perturbation on Fifteen Clustering Algorithms. Psychometrika 45: 325-342.

7. Ward JH, Jr. (1963) Hierarchical grouping to optimize an objective function. JASA 58: 236-244.

8. Martis PC, Whitsett JA, Xu Y, Perl AK, Wan H, et al. (2006) C/EBPalpha is required for lung maturation at birth. Development 133: 1155-1164.
